# Exploring Dietary Salt Knowledge, Attitude, and Practices among People of African Descent in the United Kingdom: A Qualitative Study

**DOI:** 10.3390/healthcare12191969

**Published:** 2024-10-02

**Authors:** Jesse Enebi Usman, Alexandra Morley, Charmaine Childs, David Rogerson, Markos Klonizakis

**Affiliations:** 1 Lifestyle Exercise and Nutrition Improvement (LENI) Research Group, Department of Nursing and Midwifery, Sheffield Hallam University, Sheffield S1 1WB, UK; 2 College of Health, Wellbeing and Life Sciences, Sheffield Hallam University, Sheffield S1 1WB, UK; 3 Sports and Physical Activity Research Centre, Sheffield Hallam University, Sheffield S1 1WB, UK

**Keywords:** dietary salt, people of African descent, public health initiatives, hypertension, cultural sensitivity, health literacy, cardiovascular diseases

## Abstract

**Background/Objectives:** People of African Descent (PoAD) in the United Kingdom (UK) are at an increased risk of hypertension and cardiovascular disease (CVD), partly due to dietary habits such as high salt intake. This study sought to understand the dietary salt-related knowledge, attitudes, and practises (KAP) of PoAD in the UK, to inform the development of culturally tailored interventions to reduce dietary salt intake in this population. **Methods:** We collected data on KAP from 21 PoAD across various regions in the UK through online semi-structured interviews and analysed them using reflexive thematic analysis (TA). **Results:** The age of the participants ranged from 20 to 70 years (43 ± 11). Six overarching themes were identified: (i) the multifaceted roles of salt in culinary practises, (ii) the increased awareness of health risks associated with high salt intake, (iii) the existence of knowledge gaps regarding recommended daily salt intake, (iv) the cultural influences on salt consumption levels, (v) the lack of engagement with food labels, and (vi) a limited awareness of salt reduction initiatives. **Conclusions:** Our findings highlight the significance of salt in the culture and culinary practises of PoAD. Despite general awareness of the health risks of excessive salt consumption, there was a notable deficiency in knowledge about the recommended salt intake levels as well as minimal engagement with nutritional labelling. These findings underline a need for culturally sensitive health interventions that integrate culinary practises, beliefs, and preferences of PoAD, aiming to effectively reduce salt intake and mitigate associated health risks.

## 1. Introduction

Cardiovascular diseases (CVDs) remain a major public health challenge in the United Kingdom (UK), consistently ranking as a leading cause of mortality and morbidity [1]. Currently, an estimated 7.6 million people in the UK live with CVDs, which incurs an annual direct cost of approximately GBP 9 billion to the health and social care system [2]. The overall economic impact on the UK, including costs related to premature death, disability, and informal care, was around GBP 19 billion in 2015. With CVD prevalence continuing to rise amidst declining mortality rates, these costs are expected to increase [1,2]. People of African Descent (PoAD)—encompassing those of Black African or Afro-Caribbean backgrounds—are at an elevated risk of developing CVDs [3]. This increased risk is largely attributed to a higher prevalence of primary CVD risk factors among PoAD, such as hypertension and type 2 diabetes [4]. It has been suggested that the prevalence of hypertension among PoAD in the UK is higher than that of the white population [5,6]. Additionally, PoAD exhibit a higher rate of hypertension and associated health complications, such as stroke and end-stage kidney diseases [3]. This demographic tends to have elevated blood pressure from a young age, with a consistently higher occurrence and frequency of hypertension throughout their lives [7]. Ho et al. [8] suggest that national healthcare and social policies should adopt an ethnicity-specific approach towards CVD risk factors, as such targeted strategies are crucial for reducing the overall CVD incidence and burden. Examples of these tailored approaches could include culturally sensitive public health initiatives, community-based interventions, and personalised healthcare plans that consider the unique health needs and cultural nuances of PoAD.

A high-salt diet can lead to fluid retention in the body, causing an increase in blood volume and blood pressure [9,10]. This elevated blood pressure can damage the heart and blood vessels over time, resulting in CVDs [11,12]. Furthermore, a high sodium diet is linked to several physiological changes, including water retention, increased vasomotor tone that leads to increased vascular resistance in the peripheral vasculature, changes in the function of blood vessel walls, and altered nervous system activities that affect the cardiovascular system [13,14]. Cultural dietary practises and preferences within this community often involve foods with higher salt content, further exacerbating risks in this population [15]. Physiologically, some PoAD have been found to have increased salt sensitivity, a condition where blood pressure is more reactive to salt intake [14]. The heightened salt sensitivity observed in some PoAD is a result of intricate interactions between genetic, epigenetic, environmental, and social determinants of health [14,16,17]. Genetic variations linked to the transport and metabolism of sodium can affect the body’s ability to process excess salt. Furthermore, epigenetic mechanisms such as DNA methylation and environmental influences, including diet and lifestyle choices, contribute to the risk. Social determinants of health, such as socioeconomic status and living conditions, also play important roles [16,17].

The World Health Organisation (WHO) recommends that adults limit their daily salt intake to less than 5 g to lower the risk of hypertension and CVDs. However, the global average daily salt intake for adults is 10.78 g (equivalent to 4310 mg of sodium per day), resulting in an estimated 1.89 million deaths annually [18]. To address the issue of excessive salt consumption, the UK government has launched several initiatives. Notably, in the early 1990s, the Council on Medical Aspects of Food and Nutrition Policy (COMA) recommended a reduction in the national average salt intake to 6 g/day for adults, a guideline further supported by the Scientific Advisory Committee on Nutrition (SACN) in their 2003 “SACN Salt and Health” report [19,20]. Building on previous targets, Public Health England (PHE), now succeeded by the UK Health Security Agency and Office for Health Improvement and Disparities, set forth new targets to gradually reduce the salt level in high-salt foods by 2024 [21]. Despite these initiatives, Song et al. [22] highlight a concerning trend reversal: a decline in salt intake from 9.38 g/day in 2003 to 7.58 g/day in 2014, followed by an increase to 8.39 g/day by 2018. According to the most recent National Diet and Nutrition Survey (NDNS), the current average dietary salt intake among adults in the UK is 8.4 g/day, thus underscoring a pressing need for more effective public health measures [23].

Reducing salt intake is essential for public health and economic efficiency, as it can significantly improve health outcomes. The WHO estimates that every USD 1 invested in sodium reduction initiatives yields a return of at least USD 12 [18]. The British Heart Foundation [24] reported that a reduction of just one gramme of salt per day in the UK diet could prevent 4147 deaths and non-fatal incidents annually and save up to GBP 11.4 billion by 2035. Acknowledging the importance of this issue, the WHO set a global objective of reducing salt intake by 30% by 2025 [18]. Aligning with the objectives of the WHO, this study explored the KAP related to dietary salt among PoAD in the UK. The findings were intended to inform the development of culturally tailored interventions that address the disproportionate burden of hypertension and related diseases among PoAD. This effort supports both global health initiatives and the specific needs of the population.

## 2. Methods

### 2.1. Sampling and Recruitment Process

The study targeted UK residents of African and Afro-Caribbean descent aged 18 years or older who can navigate internet-based devices. Participants were selected using a purposive sampling strategy, ensuring that those recruited had characteristics relevant to the research question. Purposive sampling is a non-probability sampling method that is commonly used in qualitative research [25]. This method involves selecting participants who have specific characteristics and qualities that enable a detailed exploration of a research question [26]. Our selection criteria did not aim to guarantee the randomness of our sample, but to obtain in-depth and nuanced insights from different demographics and geographical locations within the UK.

The recruitment channels included a variety of sources, such as open email invitations and word-of-mouth invitations, as well as the use of social media platforms (Facebook and LinkedIn). It was ensured that only individuals who met our predetermined eligibility criteria were recruited. In addition, it was a requirement that all participants provide written informed consent before taking part in the study. This ensured that the participants were well-informed about the study and its objectives and were willing to participate in the study on their own accord.

### 2.2. Eligibility Criteria

- Age: Must be 18 years or older.
- Ethnic background: individuals of African descent.
- Residence: currently residing in the UK.
- Language: English.
- Health: generally in good health, without chronic conditions that can hinder participation in the study.
- Cognitive ability: participants must be able to read, understand, and provide written informed consent based on the participant information sheet.
- Internet literacy: competent in using online platforms and tools.

### 2.3. Ethical Approval

Ethical approval for this study was obtained from Sheffield Hallam University’s Ethical Committee (Ethic Review ID: ER45058101). Key ethical aspects of this research involve ensuring voluntary participation, safeguarding participant rights, and maintaining strict confidentiality.

### 2.4. Data Collection

The interviews were conducted online via Zoom^®^ [27,28] over eight weeks, from October to December 2022. Each session lasted between 30 and 60 min. An interview guide was developed after an extensive literature review and consultations with PoAD [29]. The interview guide focused on key themes related to participants’ KAP regarding dietary salt intake. These themes included awareness of health risks, understanding of recommended daily intake, cultural factors influencing salt use, engagement with food labels, and awareness of public health efforts. The interviews were conversational in nature to encourage detailed discussions. With the consent of the participants, the interviews were recorded and transcribed verbatim [30].

### 2.5. Data Analysis

This study utilised reflexive thematic analysis (TA), as outlined by Braun and Clarke [31], to explore the KAP related to dietary salt consumption among PoAD in the UK. We selected reflexive TA as our method due to its flexibility and depth, which allow an in-depth investigation of qualitative data. In this approach, the researcher plays an active role in interpreting the data, making it an ideal method for exploring the nuanced perspectives and experiences of PoAD with regard to their dietary salt intake.

Following the suggestion of Braun and Clarke [31,32,33], it is essential to establish various theoretical assumptions across multiple continua to guide the research. One such continuum is the distinction between essentialist and constructionist epistemologies. This study adopts the constructionist epistemology [34], which views knowledge as being co-constructed by the researcher and the participants. This approach is valuable for exploring dietary salt KAP within the cultural context of PoAD. Another key continuum is experiential versus critical orientation to data. This study takes an experiential approach [34], prioritising the capture of participants’ experiences and perceptions. This approach is crucial for gaining insights into the dietary salt KAP among PoAD. 

We also considered the continuum between inductive and deductive analysis. For this study, we adopted an inductive approach, allowing themes to emerge naturally from the data [34]. This approach helped to prevent the imposition of preconceived views onto the data and enabled the discovery of unexpected insights. Lastly, we utilised both semantic and latent coding [34]. By applying this dual coding strategy, we were able to comprehensively explore the participants’ experiences, capturing both the overt and underlying meanings of their narratives.

The data analysis process adhered to the six-phase framework of reflexive TA as outlined by Braun and Clarke [32,33] and further elaborated by Bryne [34]. Through repeated readings, we identified initial codes and themes. The data were coded systematically, focussing on extracting meaningful units of analysis. The initial codes were grouped into potential themes, which were further refined and validated to ensure they accurately represented the data and captured the core aspects of participants’ salt-related KAP. Finally, the themes were defined, named, and integrated into the analytical report [34].

The research process was inherently reflexive, with the researcher documenting their reflections throughout the analysis to enhance interpretative depth and mitigate personal biases. The findings were subjected to a careful review process by the co-authors to refine the report and challenge assumptions while being grounded in the relevant literature and theoretical frameworks.

### 2.6. Reflexivity and Positionality

The leading author, as a person of African descent and a public health and mental health nursing academic, was able to understand the cultural nuances that influence dietary behaviours among PoAD in the UK. However, it was recognised that his experiences and biases might shape the research outcomes, especially as he acted as an interpreter of cultural narratives and a mediator between the lived experiences of the PoAD and the academic and policy-making audiences. To ensure that the study’s constructs are grounded in the participants’ narratives rather than being influenced by preconceptions or biases, the research practise was deeply influenced by a commitment to reflexivity. This meant scrutinising all interactions with the research materials, the participants, and the broader thematic analysis process.

### 2.7. Sample Size Determination

Determining sample size in reflexive TA necessitates a change from the traditional concepts of data saturation to a more nuanced and flexible approach based on the idea of information power. This approach suggests that a smaller sample size may be sufficient if the data it holds is highly relevant to the study’s objectives [35]. Reflexive TA emphasises that the meaning of data is not predetermined but rather emerges from the researcher’s active interaction with it [34]. Consequently, new insights may emerge as long as data analysis continues [36], making it challenging to define a specific point at which data saturation is reached [35]. In this study, our sample size of 21 was guided by the concept of information power, aligning with the broader understanding in qualitative research that reflects the dynamic and interpretative nature of reflexive TA [35,37].

## 3. Results

### 3.1. Demographic Characteristics of Participants

This qualitative study gathered demographic data from 21 participants (Table 1). The group comprised 67% females (14 out of 21) and 33% males (7 out of 21). All participants identified as having African or Afro-Caribbean heritage, offering culturally relevant insights. Participants’ ages ranged from 20 to 70 years, with an average age of 43 (±11.4) years. The wide age range was purposefully selected to ensure a comprehensive understanding of dietary salt KAP, recognising that dietary behaviours and health awareness can shift significantly with age [38,39]. Capturing diverse perspectives is critical to fully understanding the risk factors associated with excessive salt intake and how they may manifest differently based on where individuals are in their life course.

**Figure 1:**
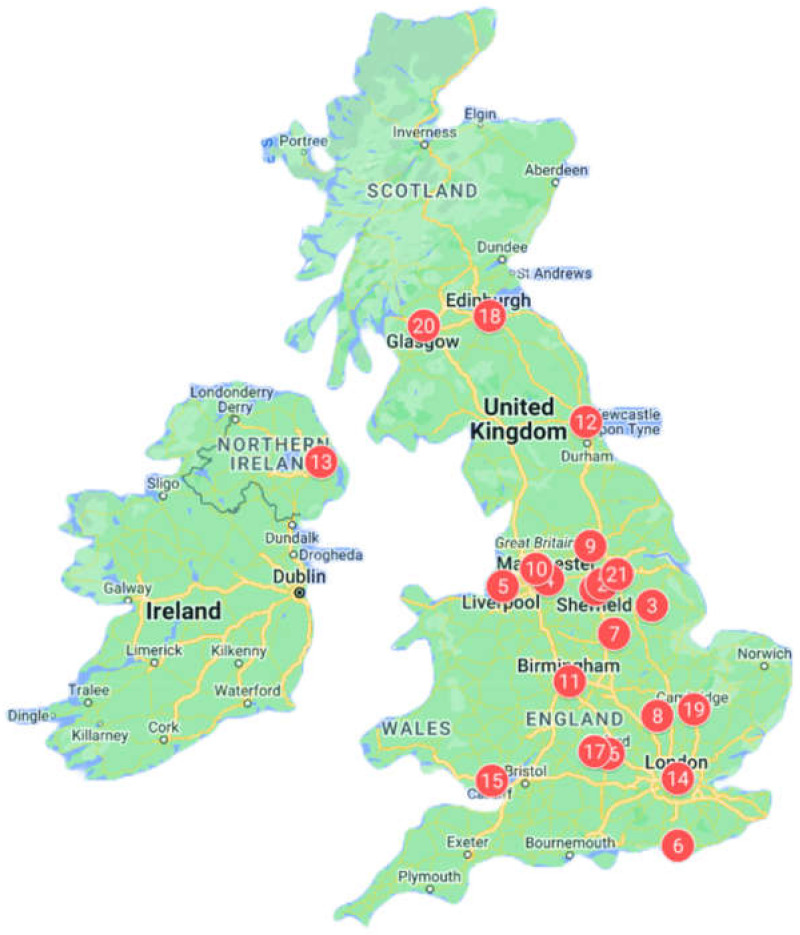
Geographical distribution of participants across various locations in the UK. The red dots represent the participant locations, and the numbers indicate the order in which the interviews were conducted. Map data @2024 Google.

Furthermore, participants were purposively recruited from a broad geographical location in the UK. Participants were selected from 21 different urban and suburban areas in the UK, including cities like Manchester, Cardiff (Wales), Belfast (Northern Ireland), Leeds, Sheffield, London, Glasgow (Scotland), and Birmingham. Figure 1 illustrates the geographical distribution of the participants across various locations in the UK. Additionally, the ethnic composition of the participants also widely varied, with 52% (11/21) identifying as African and 48% (10/21) as Afro-Caribbean. This diversity ensured a well-balanced participant pool and allowed for an in-depth exploration of dietary salt KAP across the population.

### 3.2. Selection of Participant

**Figure 2:**
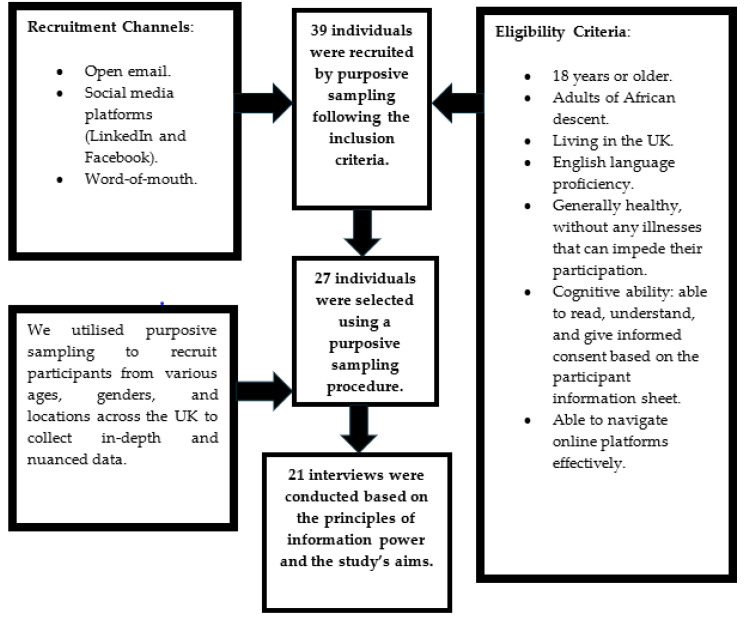
Eligibility criteria, recruitment strategies, and selection process.

We initially recruited 39 adults of African descent who met our inclusion criteria. The sample had a gender ratio of approximately 1:2 (male:female), indicating a higher response from female participants to our invitation. After the initial recruitment, 27 participants were selected for their potential to offer diverse and nuanced perspectives, as presented in the participant selection flowchart (Figure 2). This methodological approach was crucial in ensuring that the study captured a wide range of insights, thereby enhancing the research findings [26].

A total of 21 interviews were conducted, guided by the principles of information power [35] and incorporating the interpretative and reflexive nature of TA [37]. This number was determined based on our judgement of the richness and scope of the data in addressing our research question, reflecting a pragmatic approach to sampling within the limits of our study [36,40].

### 3.3. Identified Themes

Six overarching themes emerged from our reflexive TA of interview transcripts with participants. Table 2 provides a summary of these themes, which encompass the complex ways in which salt is used in culinary practises, the level of awareness of health risks associated with high salt intake, knowledge gaps regarding recommended daily salt intake, cultural influences on salt consumption levels, minimal engagement with food labels, and limited awareness of online salt reduction resources.

#### 3.3.1. Theme 1: The Multifaceted Roles of Salt in Culinary Practises

In exploring participants’ perspectives on salt and its various applications, a recurring theme emerged, highlighting salt’s integral role in culinary practises. All respondents agreed on the significance of salt as a fundamental component in their cooking, highlighting its universal presence in their dietary habits.

Several participants repeatedly emphasised the flavour-enhancing properties of salt: “*I know salt makes food taste nice, so it is a very important ingredient when cooking most of the time*” (Participant 6, female, 51, African), and “*salt is an essential ingredient in food. It gives food it taste*” (Participant 21, male, 49, Afro-Caribbean). These statements reflect a deep-seated appreciation for salt’s role in advancing food taste. One participant highlighted an unconventional yet culturally significant usage, stating, “*We also use salt to wash our vegetables*” (Participant 1, female, 27, Afro-Caribbean), exemplifying salt’s diverse utility and hints at traditional practises embedded within certain communities. 

Furthermore, the study revealed salt’s significance extending beyond taste enhancement to preservation techniques, particularly in African culinary traditions. As one participant noted, “*Salt is a tasty ingredient that we use in cooking, not just in cooking, also in preservation… so salt is one of the ingredients that we use in cooking…*” (Participant 10, female, 70, African). This highlights salt’s versatile role in various food preparation methods, including preservation and drying, which are integral to maintaining food quality and longevity. Another participant stated, “*I like salt. I doubt if our people can make any meal without salt*” (Participant 13, male, 20, African). This captures the collective view of salt as an indispensable component in culinary practises, essential for its flavour-enhancing properties and its functional roles in food preservation and preparation.

#### 3.3.2. Theme 2: Awareness of the Health Risks Associated with High Salt Intake

This theme explores participants’ understanding of the health implications of high salt intake. Among the respondents, about half (10/21) believed that consuming too much salt is unhealthy. Our analysis also revealed a range of views about the negative health effects of high salt consumption. One participant acknowledged the personal risk and the need for action: “*I know too much salt is not good for me and I know later in future it may affect me. I have been eating salt knowing what salt can cause, I think it is better for me to intervene now and reduce the amount of salt I eat*” (Participant 2, female, 39, Afro-Caribbean). Echoing this sentiment, another participant (Participant 17, male, 36, Afro-Caribbean) shared his personal struggle with reducing salt usage due to cultural expectations: “*Salt is an ingredient, but I know it is bad for our health. I was kind of trying to avoid cooking with salt in my house, but when I cook meals like that, people tend to go and add salt, and this is like a common culture among the blacks”.* This narrative suggests the challenge of modifying deeply rooted dietary habits within the African diaspora, highlighting the tension between health knowledge and cultural practise.

Another respondent highlighted specific health concerns: “*…too much of it can be detrimental to the health. I know it is detrimental to kidneys*” (Participant 6, female, 51, African), indicating awareness of the potential for organ damage. Further, the impact of traditional food preservation methods involving salt was noted: *“We’ve got some things that we preserve with salt, for example, we’ve got the locally made dried catfish. We use salt already to preserve it, so just imagine using the same catfish to cook another meal, and still adding salt… it’s definitely going to be bad for our health*” (Participant 10, female, 70, African). 

Several participants explicitly recognised the link between salt intake and hypertension. One respondent stated, “*…because it is unavoidable that is why we have these high cases of high blood pressure*” (Participant 1, female, 27, Afro-Caribbean), and “*There is a relationship between the excessive consumption of salt and hypertension*” (Participant 21, male, 49, Afro-Caribbean). 

#### 3.3.3. Theme 3: Knowledge Gaps Regarding Recommended Daily Amount of Dietary Salt 

During the interviews, participants were asked about their knowledge of the recommended daily salt intake. The majority (18/21) were unaware of any specific guidelines. After being informed of the WHO recommendation of less than 5 g (just under a teaspoon) of salt per day, many participants acknowledged that their salt consumption exceeded this recommendation. 

Some participants expressed their limited knowledge and awareness of the recommended amount of daily salt intake: “*No, I don’t. I have no idea at all*” (Participant 7, male, 49, African), and “*I wouldn’t say, I know*” (Participant 11, male, 51, African), highlighting a common theme of awareness of the need for moderation without specific knowledge of the recommended guidelines.

One participant, uncertain about the exact figures, admitted, “*Specifically, I don’t know the average. Just know it should not be too high because of the high levels of risks*” (Participant 1, female, 27, Afro-Caribbean). This statement indicates a recognition of the need to limit salt intake, albeit without precise knowledge of the guidelines.

Participant 18 (female, 43, African) stated, “*I probably eat too much salt, but I’m not really sure how much is supposed to be safe”.* Another respondent, who appeared to try recalling the guideline, mentioned, “*The last time I checked, we are allowed one or two teaspoons or a full spoon of salt daily, but I eat a lot of salt daily*” (Participant 2, female, 39, Afro-Caribbean), indicating some awareness but also uncertainty about the exact recommended amount. A similar sentiment was echoed by another participant: “*To the best of my knowledge, I think a spoon of salt is what’s recommended. I think so though not very sure*” (Participant 10, female, 70, African), further illustrating the prevalent uncertainty regarding the guidelines.

Please note: The interviewer asked follow-up questions to clarify any ambiguities during the interviews. This approach was consistent across all interviews to ensure a standardised process for gathering accurate information. For example, when Participant 10 mentioned, ‘I just use a spoon of salt,’ the interviewer asked, ‘Could you please specify what type of spoon you are referring to?’ Participant 10 clarified that she was referring to a teaspoon.

#### 3.3.4. Theme 4: Cultural Influences on Dietary Salt Consumption

The cultural influence on salt consumption was a prominent theme, with many interviewees agreeing that PoAD generally consume more salt than other ethnic groups. “*We definitely consume more salt than other ethnicities. We like to eat freshly cooked meals and most of the time we use salt to cook*”, shared one participant (Participant 1, female, 27, Afro-Caribbean). Another participant added, “*…our culture affects our salt intake because generally we like salt in our diet and salt is found in different forms such as our seasoning cubes and some spices, and our appetite is developed based on that*” (Participant 8, male 28, Afro-Caribbean). 

Participants also commented on the prevalence of salt in African culinary traditions: *“Africans do eat a lot of salt. Okay*” (Participant 11, male, 51, African). The cultural contrast was highlighted by a respondent who observed, “*Blacks like lots of salt, pepper, and spicy stuff. This is as opposed to the whites who do not do lots of spices and salts*” (Participant 19, female, 60, African). One participant noted, “*I think black people consume more salt than any other ethnicity. There is hardly a place you visit that you won’t find salt in their meals*” (Participant 7, male, 49, African). 

Among participants with hypertension or a family member with a history of the condition, we observed a cautious approach to salt consumption. Participant 8 (male, 28, Afro-Caribbean) remarked, “*My family doesn’t eat much salt because my dad had high blood pressure and told us that salt was the cause*”, illustrating how health concerns shape dietary practises. Similarly, Participant 21 (male, 49, Afro-Caribbean) noted, “*Since I have high blood pressure, I only eat a small amount of salt*”. This highlights the direct impact of personal health issues on dietary salt intake.

#### 3.3.5. Theme 5: Minimal Engagement with Food Labels

The study delved into the habits of the participants concerning reading food labels. It was found that a limited number of respondents (5/21) engaged in reading food labels, primarily focussing on expiry dates and allergens other than salt content. Four (4/21) check expiry dates of food before purchasing them, while one participant (1/21) reads food labels to check for allergens. 

One participant stated: “*My wife does most of our shopping and cooking anyway, so I hardly buy food items. When I buy food, I never read any other thing apart from the expiry date. That’s my major concern*” (Participant 4, male, 54, Afro-Caribbean). Similarly, participant 15 (female, 47, African) responded: “*not all the time. I examine the expiry dates of food items before I buy them*”. These comments highlight a general trend among participants to prioritise checking expiry dates over other information on food labels. We also found that specific health concerns dictated the label-reading habits of one participant, stating, “*I am allergic to a few things, so I check for allergens*” (Participant 17, male, 36, Afro-Caribbean). 

#### 3.3.6. Theme 6: Limited Awareness of Salt Reduction Initiatives

This theme examines participants’ awareness of public health campaigns or initiatives to reduce salt intake. Only one respondent indicated awareness of a specific campaign focused on salt and sugar reduction. The sole participant who was aware of such a campaign mentioned, “*I know of one website that promotes salt and sugar reduction. I looked up information on salt reduction on the internet after my father told me about the link between salt and hypertension*” (Participant 8, male, 28, Afro-Caribbean). This statement indicates a personal motivation for seeking information driven by familial health concerns. Another participant shared a poignant personal reason for wanting to reduce her salt intake: “*my mum passed away some years ago… she had hypertension. I want to adhere to the rules but I do not know any source to get regular updates or information. I want to live healthy and longer*” (Participant 16, female, 45, African). This addition highlights a common desire among participants to improve their health but reveals the paucity of accessible information on how to achieve this. 

## 4. Discussion

Our exploratory study on the dietary salt KAP among PoAD in the UK has uncovered a complex interplay of behavioural, cultural, and health literacy factors that influence dietary salt consumption. Although existing literature provides a background for understanding these dynamics, our study contributes fresh insights to the nuanced perspectives of PoAD on salt consumption and its effects on health.

### 4.1. Gender Distribution and Salt Consumption

Our study presents a gender ratio of approximately 2:1 (female: male), indicating a higher response from females to our invitation. This gender disparity may be attributed to an increased readiness by women to participate in research [41,42]. Potential explanations for this discrepancy are multi-faceted and may include higher health consciousness or varying relevance as perceived by women [43]. Gender plays a crucial role in shaping dietary habits and health outcomes, influenced by a complex interplay of biological, social, and cultural factors [44]. For example, women may have different nutritional needs and health concerns as compared to men, which could cause their patterns of salt intake and engagement with health information about salt consumption to be different. 

These gender-specific findings highlight the need to integrate gender issues into public health interventions aimed at reducing high salt intake. Tailoring strategies could involve empowering women with knowledge and skills to reduce salt usage in cooking. For men, interventions may focus on highlighting the direct consequences of dietary choices on personal health. This could be through digital platforms or community programmes that can effectively engage male audiences [41].

### 4.2. Ethnic Backgrounds and Dietary Behaviours

We compared the salt-related KAP of African and Afro-Caribbean participants. Despite our expectation of differences in their dietary salt KAP, mostly due to environmental and cultural factors, they showed a remarkable similarity. This suggests that shared cultural practises may play a more significant role in shaping dietary behaviours than distinct ethnic traditions [45]. Our study underscores the need to consider the broader cultural context that includes both African and Afro-Caribbean communities. It is also essential to recognise the complexity of cultural identity and its potential influence on health behaviours. While our study offers a foundational understanding of the similarities in salt-related dietary practises between these communities, it also highlights the necessity for further investigation. A deeper exploration into the nuanced differences between African and Afro-Caribbean populations could reveal valuable insights that were beyond the scope of this study. 

### 4.3. Cultural Influences on Salt Consumption Patterns

Our research sheds light on the important role that salt plays in the culinary and cultural traditions of the PoAD. In addition to being a flavour enhancer, salt is also used in traditional food preservation methods and for washing vegetables. Our participants explained how this practise is an important part of their cultural identity and how it shapes their dietary habits. This perspective is supported by previous research that highlights the central role of culinary traditions in shaping dietary behaviours [15,46]. The dual role of salt, both as a flavour enhancer and a preservative, highlights the need for nuanced public health interventions. Promoting the reduction in salt intake is important, but any initiative should be carried out in a way that considers the cultural, social, and historical contexts of the target population.

We found that culture and tradition play a significant role in determining salt consumption levels among PoAD in the UK. Participants expressed a strong emotional attachment to culinary practises that heavily feature salt for flavour enhancement and preservation. This cultural affinity poses a significant challenge for public health interventions aimed at reducing salt intake without separating the community from its cultural roots. Our findings are consistent with previous research by Rousham et al. [46], which highlights the diversity and cultural specificity of dietary patterns among PoAD. The deeply ingrained preference for salt in culinary traditions necessitates an approach that respects these practises while guiding individuals towards healthier choices. This could be achieved by developing low-sodium versions of popular seasonings [47,48] or by organising workshops on alternative cooking techniques that enhance the flavour of food [49] without relying heavily on salt. Another strategy that might be useful is the involvement of community leaders and influencers who can be instrumental in advocating for healthier dietary practises. Engaging respected individuals within PoAD communities to model and endorse reduced-salt cooking can have a positive ripple effect, promoting healthier choices within the community [50].

### 4.4. Improving Awareness and Engagement with Nutritional Information

Our study revealed an important discrepancy. While the participants were aware of the health risks associated with a high-salt diet, they lacked knowledge about specific dietary guidelines or recommendations for salt intake. This clearly indicates the urgent need to improve health literacy in a culturally relevant manner, which is pivotal in bringing about changes in behaviour [51]. To fill this knowledge gap, our findings suggest the importance of targeted educational campaigns that not only communicate the health risks of excessive salt intake but also provide clear and accessible guidance on recommended intake levels. These campaigns should incorporate the unique cultural and social dynamics of the PoAD, ensuring that messages are relevant and engaging. 

We also found that many PoAD in the UK do not read food labels. This serves as a major hindrance to informed dietary salt reduction decision-making. To address this knowledge gap, public health initiatives should consider introducing simplified labelling systems that present key nutritional information in a visually engaging manner. The traffic light system, which categorises nutritional content with colour coding, has shown promise in helping consumers make healthier food choices by providing at-a-glance information [52]. This approach could be particularly useful for PoAD, making it easier to quickly assess the salt content of foods.

The complexity of nutritional labelling has been identified as one of the obstacles deterring consumers from utilising these labels effectively to guide their dietary choices [53,54]. This problem is further exacerbated when there is a lack of awareness regarding the health implications of high salt intake. The limited attention paid by participants to nutritional labels, especially salt content, highlights the need for interventions to create awareness and make such information more accessible, understandable, and relevant to this community.

Digital technology presents largely unexplored opportunities for enhancing nutritional literacy among PoAD. Recent studies have shown that digital health interventions can be effective in improving dietary habits and health literacy across diverse populations [55]. For example, social media campaigns, web-based health education, and digital technologies can be used to provide simplified nutritional information and suggest healthier options that cater to the specific dietary needs and preferences of PoAD [56]. Exploring these digital solutions further could help to overcome the unique challenges faced by PoAD in cultivating healthy eating habits and improving their overall health and well-being.

### 4.5. Study Strengths

The study incorporates several strengths contributing to its credibility and transferability. The diverse sampling technique ensured the inclusion of individuals from various regions across the UK and spanning different generations [57]. Similar to the approach noted in other research [58], conducting online interviews as a data collection method allowed for the inclusion of participants from various locations across the UK. This approach offered flexibility in scheduling and significantly expanded the reach of the study. The study’s participant selection strategy was developed to ensure that we capture a wide spectrum of the perspectives of PoAD. The diverse participant pool strengthens the generalisability of the findings of our study [59], ensuring that the insights gained reflect the variety of experiences and standpoints of dietary salt-related KAP among PoAD. The study employed reflexive TA to facilitate a comprehensive exploration of complex factors influencing dietary salt KAP among PoAD. This method encourages researchers to critically engage with the data while reflecting on their role and involvement in the research process [60], thereby enhancing the validity and credibility of the findings [34]. The broad age range ensures that the study captures diverse perspectives of PoAD, reflecting the variations at different life stages within the community. This approach aligns with previous studies emphasising the importance of age diversity in health-related studies [61,62].

### 4.6. Study Limitations

Focussing on participants who are active internet users and possess the necessary technological devices and skills may have potentially limited the diversity of our sample. Despite efforts to engage a wider representation of the PoAD in the UK, the sample is skewed towards individuals who are educated. Therefore, our findings should be understood within this context, taking into account the intersectionality within ethnic groups. Another limitation to consider is social desirability bias, where participants may have given responses that appear more socially acceptable or aligned with the interviewer’s perceived expectations [63]. To reduce this risk, participants were assured of the confidentiality of their responses and the importance of providing honest feedback was emphasised. The absence of face-to-face interviews, a limitation acknowledged in previous studies [64], may have restricted the ability to capture non-verbal cues and subtle nuances. However, the interviewer compensated for this by paying close attention to vocal cues and inflexions during the online interviews. Finally, a further limitation of this study is the lack of triangulation with key informants or observational techniques, especially for behaviours like food label reading, which may not be fully captured by self-reports. As noted by Denzin [65], triangulation enhances the robustness of qualitative research by validating findings from different sources. While this study relied on self-reported data, future studies could incorporate observational methods and input from public health professionals and cultural leaders to provide a more comprehensive understanding of dietary salt practises among PoAD in the UK.

## 5. Conclusions

Our investigation of the dietary salt KAP among PoAD in the UK has unveiled valuable insights into the cultural, behavioural, and health literacy factors that influence salt consumption patterns. The study’s findings highlight the significant role of gender, showing a preference by female participants towards research engagement, most likely reflecting broader societal trends in health consciousness and dietary management. This gender dynamic suggests the need for gender-sensitive strategies for tailored public health strategies, addressing dietary behaviours and health outcomes.

Our research identified the cultural underpinnings of dietary salt consumption as a significant theme, revealing a deep-rooted tradition of salt usage among PoAD. Although this cultural affinity showcases the rich culinary heritage, it presents unique barriers to public health efforts aimed at dietary salt reduction. Our study emphasises the need for culturally sensitive interventions that respect and integrate the values and practises of PoAD, advocating for a collaborative approach to developing acceptable and effective dietary guidance.

The study identified gaps in knowledge about recommended daily salt intake and minimal engagement with food labels, signalling the urgent need for improved health literacy. We recommend targeted educational campaigns and innovative labelling strategies that resonate with the cultural and social realities of PoAD, aiming to bridge the information gap and empower individuals with the knowledge to make informed dietary choices. In addition, the limited awareness of salt reduction initiatives among our participants indicates a critical opportunity for public health professionals to increase their outreach and engagement efforts. Leveraging digital platforms and community networks to disseminate vital messages and create a supportive environment for healthy dietary choices is necessary.

Finally, our comprehensive analysis and diverse participant pool provide valuable insights into the complex interplay of factors influencing dietary salt KAP among PoAD in the UK. The study underscores the importance of adopting a multifaceted and culturally attuned approach in public health interventions to effectively address the challenges of high salt consumption and associated health risks within this community. It is imperative that future research and public health strategies consider the nuanced perspectives and needs of PoAD, fostering an inclusive and equitable pathway towards improved dietary health and well-being.

For the purpose of open access, the author has applied a Creative Commons Attribution (CC BY) licence to any Author Accepted Manuscript version arising from this submission.

## Figures and Tables

**Table 1:** Demographic characteristics of respondents.

Variable		N (%)
Gender	Female	14 (67)
	Male	7 (33)
Ethnicity	African	11 (52)
	Afro-Caribbean	10 (48)
Age range (years)	20–29	3 (14)
	30–39	4 (19)
	40–49	7 (33)
	50–59	3 (14)
	60–69	3 (14)
	70+	1 (5)

**Table 2:** Summary of overarching themes identified from thematic analysis.

Theme	Description	Representative Quote
The multifaceted roles of salt in culinary practises	PoAD use salt to enhance flavour, wash vegetables, and preserve food.	“Salt is a tasty ingredient that we use in cooking, not just in cooking, also in preservation…so salt is one of the ingredients that we use in cooking…” (Participant 10, female, 70, African).
Awareness of health risks associated with high salt intake	Participants are aware of the health risks associated with excessive salt consumption, including hypertension and kidney damage, but cultural practises often override these concerns.	“I know too much salt is not good for me and I know later in future it may affect me. I have been eating salt knowing what salt can cause, I think it is better for me to intervene now and reduce the amount of salt I eat” (Participant 2, female, 39, Afro-Caribbean).
Knowledge gaps regarding recommended daily salt intake	There is a notable deficiency in knowledge about the recommended level of daily salt intake, many of the participants are unsure of the specific guidelines.	“Specifically, I don’t know the average. Just know it should not be too high because of the high levels of risks” (Participant 1, female, 27, Afro-Caribbean).
Cultural influences on salt consumption levels	Cultural practises and preferences influence high salt consumption among PoAD, which often exceeds the recommended levels.	“I think black people consume more salt than any other ethnicity. There is hardly a place you visit that you won’t find salt in their meals” (Participant 7, male, 49, African).
Minimal engagement with food labels	Participants show limited engagement with food labels, with a few participants primarily focussing on expiry dates and allergens rather than salt content.	“My wife does most of our shopping and cooking anyway, so I hardly buy food items. When I buy food, I never read any other thing apart from the expiry date. That’s my major concern” (Participant 4, male, 54, Afro-Caribbean).
Limited awareness of salt reduction initiatives	Participants have minimal awareness of internet-based resources and initiatives aimed at reducing dietary salt intake.	“My mum passed away some years ago… she had hypertension. I want to adhere to the rules, but I do not know any source to get regular updates or information. I want to live healthy and longer” (Participant 16, female, 45, African).

## Data Availability

Data are contained within the article.
